# The BONEBRIDGE active transcutaneous bone conduction implant: effects of location, lifts and screws on sound transmission

**DOI:** 10.1186/s40463-020-00454-1

**Published:** 2020-08-10

**Authors:** Seyed Alireza Rohani, Mandolin Li Bartling, Hanif M. Ladak, Sumit K. Agrawal

**Affiliations:** 1 Department of Otolaryngology – Head and Neck Surgery, Western University, London, Canada; 2 Schulich School of Medicine and Dentistry, Western University, London, Canada; 3 Department of Medical Biophysics, Western University, London, Canada; 4 Department of Electrical and Computer Engineering, Western University, London, Canada; 5 National Centre for Audiology, Western University, London, Canada; 6 Cochlear Implant Program, London Health Sciences Centre, London, Canada

**Keywords:** Bone conduction implants, BONEBRIDGE, Laser Doppler vibrometry, Conductive hearing loss, Mixed hearing loss, Middle fossa, Above the temporal line

## Abstract

**Background:**

The BONEBRIDGE (MED-EL, Innsbruck, Austria) is a bone-conduction implant used in the treatment of conductive and mixed hearing loss. The BONEBRIDGE consists of an external audio processor and a bone-conduction floating mass transducer that is surgically implanted into the skull in either the transmastoid, retrosigmoid or middle fossa regions. The manufacturer includes self-tapping screws to secure the transducer; however, self-drilling screws have also been used with success. In cases where the skull is not thick enough to house the transducer, lifts are available in a variety of sizes to elevate the transducer away from the skull. The objective of the present study was to investigate the effects of screw type, lift thickness, and implant location on the sound transmission of the BONEBRIDGE.

**Method:**

Six cadaveric temporal bones were embalmed and dried for use in this study. In each sample, a hole was drilled in each of the three implant locations to house the implant transducer. At the middle fossa, six pairs of screw holes were pre-drilled; four pairs to be used with self-tapping screws and lifts (1, 2, 3, and 4 mm thick lifts, respectively), one pair with self-tapping screws and no lifts, and one pair with self-drilling screws and no lifts. At the transmastoid and retrosigmoid locations, one pair of screw holes were pre-drilled in each for the use of the self-tapping screws. The vibration of transmitted sound to the cochlea was measured using a laser Doppler vibrometry technique. The measurements were performed on the cochlear promontory at eight discrete frequencies (0.5, 0.75, 1, 1.5, 2, 3, 4 and 6 kHz). Vibration velocity of the cochlear wall was measured in all samples. Measurements were analyzed using a single-factor ANOVA to investigate the effect of each modification.

**Results:**

No significant differences were found related to either screw type, lift thickness, or implant location.

**Conclusions:**

This is the first known study to evaluate the effect of screw type, lift thickness, and implant location on the sound transmission produced by the BONEBRIDGE bone-conduction implant. Further studies may benefit from analysis using fresh cadaveric samples or in-vivo measurements.

## Background

Bone conduction implants (BCIs) stimulate the cochlea through vibratory excitation of the temporal bone using an actuator [1]. Bone conduction implants are used to treat hearing loss when conventional hearing aids cannot be worn because of medical or anatomic conditions such as recurrent otitis externa, aural atresia, or unilateral hearing loss, among others [2]. There are two routes through which BCIs can stimulate the cochlea: over skin drives and direct bone drives. In general, the outcome of direct bone drives is better than over skin since soft tissue and skin dampen sound pressure at higher frequencies [3]. Direct bone drive BCIs can be further classified into percutaneous, passive transcutaneous or active transcutaneous devices.

Percutaneous BCIs were first introduced by Tjellström and his team in Sweden in the late 1970s [4]. The most commonly used percutaneous BCI is the bone anchored hearing aid (Cochlear™ Baha® System, Cochlear Ltd., Sydney, Australia) [2]. These devices directly vibrate the temporal bone through a surgically implanted osseointegrated titanium screw and a skin-penetrating abutment attached to an external fixture [3]. While the Cochlear™ Baha® System has favourable audiological outcomes, it is associated with certain disadvantages. For example, the skin-penetrating mechanism can cause possible infection, wound dehiscence, fixture loss, and/or the need for revision surgery [5]. It is also associated with a higher complication rate for pediatric patients compared to adults [5].

In comparison, transcutaneous BCIs benefit from the skin overlying the implanted device remaining intact. In passive transcutaneous BCIs such as the Baha Attract (Cochlear Ltd., Sydney, Australia) and Sophono (Sophono Inc., Boulder, CO, USA) [6], the actuator is located within an external housing and the external vibration is then transmitted transcutaneously to an implant that is covered by skin. Although passive transcutaneous BCIs do not have the percutaneous abutment and associated complications, they do require significant contact force and generate less gain than the percutaneous devices [3]. The force exerted by the sound processor may also cause pressure marks or skin pain, which has been associated with reduced device adherence [6].

Active transcutaneous BCIs, such as the BONEBRIDGE bone conduction implant (BB-BCI; MED-EL, Innsbruck, Austria) or Osia (Cochlear Ltd., Sydney, Australia) are semi-implantable such that the vibratory energy does not need to be transmitted through the skin [7, 8]. The BB-BCI system consists of two components: an internal implant housing the magnet, coil and actuator, also known as the bone-conduction floating-mass transducer (BC-FMT); and an external audio processor. Similar to cochlear implants, the external component is small and contains a microphone, processor and battery. The BB-BCI is associated with good functional outcomes through the direct vibration of the temporal bone while avoiding the complications associated with the percutaneous abutment and the gain loss associated with passive transcutaneous devices.

The transmitted vibration to the cochlea using BCIs has been widely studied in the literature using accelerometers [8–10] and laser Doppler vibrometry (LDV) techniques. A broad range of studies have focused on the effect of implant location on the transmitted sound to the cochlear promontory [11–18]. In studies of percutaneous devices, a significant effect was found when the implant was located closer to the cochlea [9]. However, similar studies of the effect of implant location have not yet been conducted for the BB-BCI or other active transcutaneous devices.

The BB-BCI is commonly implanted at the transmastoid (TM), retrosigmoid (RS) or recently-introduced middle fossa (MF) locations [1, 10, 11]. The BB-BCI comes with self-tapping screws included in the implant kit; however, due to incompatibility with most North American drills, has been shown to work successfully with self-drilling screws [6]. Furthermore, in cases where the skull is not thick enough to house the implant, lifts can be used to avoid dural compression. Lifts of various thicknesses (1–4 mm) can be positioned between the flanges of the implant and the skull surface to elevate the actuator from the dura. The effect of these modifications on the performance of the BB-BCI have not yet been studied. The objective of the present study was to investigate the effects of implant location, lift thickness, and screw type on the ex-vivo sound transmission of the BB-BCI.

## Material and methods

### Sample preparation

Six cadaveric temporal bones (two right ears and four left ears) were used in this study. All cadaveric specimens were obtained with permission from the body bequeathal program at Western University, London, Ontario, Canada in accordance with the Anatomy Act of Ontario and Western’s Committee for Cadaveric Use in Research (Approval number #19062014). Samples were received previously fixed in a 10% formaldehyde solution and dried.

On each sample, appropriate craniotomies were made in the TM, RS and MF locations to house the implant transducer. To expose the cochlear promontory, the external auditory canal was widened by removing cartilage and drilling the anterior and inferior walls. To expose the cochlear promontory for LDV measurements, the tympanic membrane and the malleus were removed as previously described [9]. To maximize the reflection from the cochlear promontory, a small (approximately 1 mm by 1 mm) piece of reflective tape was placed, centered between the round and oval windows.

### Test conditions

**Fig. 1:**
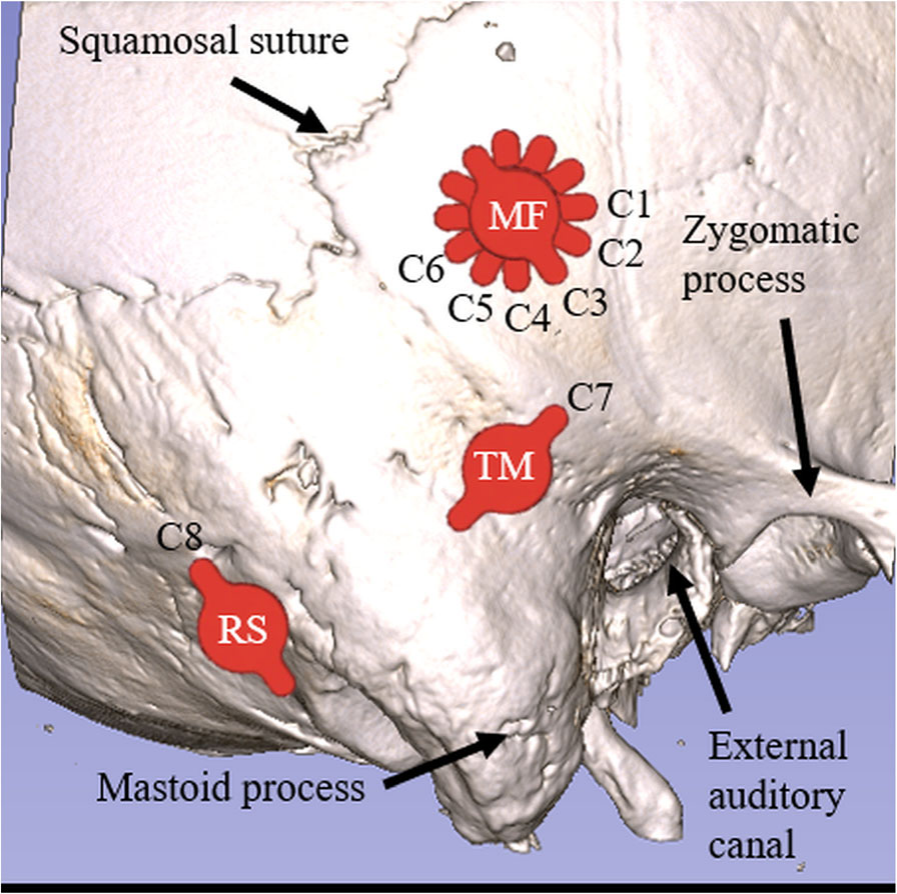
Schematic illustration of each implantation condition (C1 – C8). Six conditions were studied at the MF location, while only one condition was studied in each of the TM and RS locations. MF: middle fossa, TM: transmastoid, RS: retrosigmoid, ST: self-tapping, SD: self-drilling

Cochlear velocity was compared in eight different conditions with varying combinations of implant location (TM, RS, or MF), lift thickness (1 mm, 2 mm, 3 mm, or 4 mm) and screw type (self-drilling or self-tapping). Six conditions (C1-C6) were studied at the MF location: four with self-tapping screws and lifts (1 mm, 2 mm, 3 mm, and 4 mm thick lifts, respectively), one with self-tapping screws and no lifts, and one with self-drilling screws and no lifts. One condition (C7) was studied in the TM location with self-tapping screws and no lifts, and one condition (C8) in the RF location with self-tapping screws and no lifts. A summary of the test conditions is given in Table 1 and illustrated in Fig. 1.

**Table 1:** Location, lift thickness, and screw type for each test condition.

Condition name	Location	Lift Thickness	Screw Type
C1	MF	–	ST
C2	MF	–	SD
C3	MF	1 mm	ST
C4	MF	2 mm	ST
C5	MF	3 mm	ST
C6	MF	4 mm	ST
C7	TM	–	ST
C8	RS	–	ST

### Laser Doppler Vibrometry

For each condition, the vibration of the transmitted sound to the cochlea was measured using the LDV (Model CLV-2534, Polytec Inc., CA, USA) pointed directly on the cochlear promontory. A two degree-of-motion goniometer (Model BGS80PP and BGM120PP, Newport Corp., CA, USA) was used to accurately position the sample under the LDV device. The measurements were performed on the cochlear promontory at eight discrete frequencies (0.5, 0.75, 1, 1.5, 2, 3, 4 and 6 kHz). A 45 dB HL signal was directly transmitted to the transducer via a wired connection using the BB-BCI programming software (CONNEX, Siemens Hearing Instrument, NJ, USA). The noise vibration was also measured with no stimulation and compared with the BCI stimulated vibration. The cochlear promontory velocity was measured with an accuracy of 2 mm/s/V and is presented as frequency responses expressed in mm/s. Measurements were averaged over five repetitions.

The effect of location, lift and screw type was calculated in relative terms. Since condition C1 had two out of three common effects with the other 7 conditions (C2 to C8), it was chosen as the reference condition for analysis in relative terms, meaning the velocity of all conditions was compared to the reference and reported in dB unit.

A pilot study was performed that showed that ±5 dB HL input would change the output velocity from 0.1 mm/s to 0.13 mm/s with a standard deviation of 0.02 mm/s. Based on that study, a sample size calculation was performed to confirm that 6 samples were needed to provide a statistical power of 80% to find the equivalent of a ± 5 dB HL difference. A one-way analysis of variance (ANOVA) was used to compare the effects of variations between location, lift thickness and screw type at each frequency. Bonferroni correction was used to account for multiple testing (i.e., at 8 frequencies); therefore, the *p* < 0.006 was used to control the familywise error rate at *p* ≤ 0.05.

## Results

**Fig. 2:**
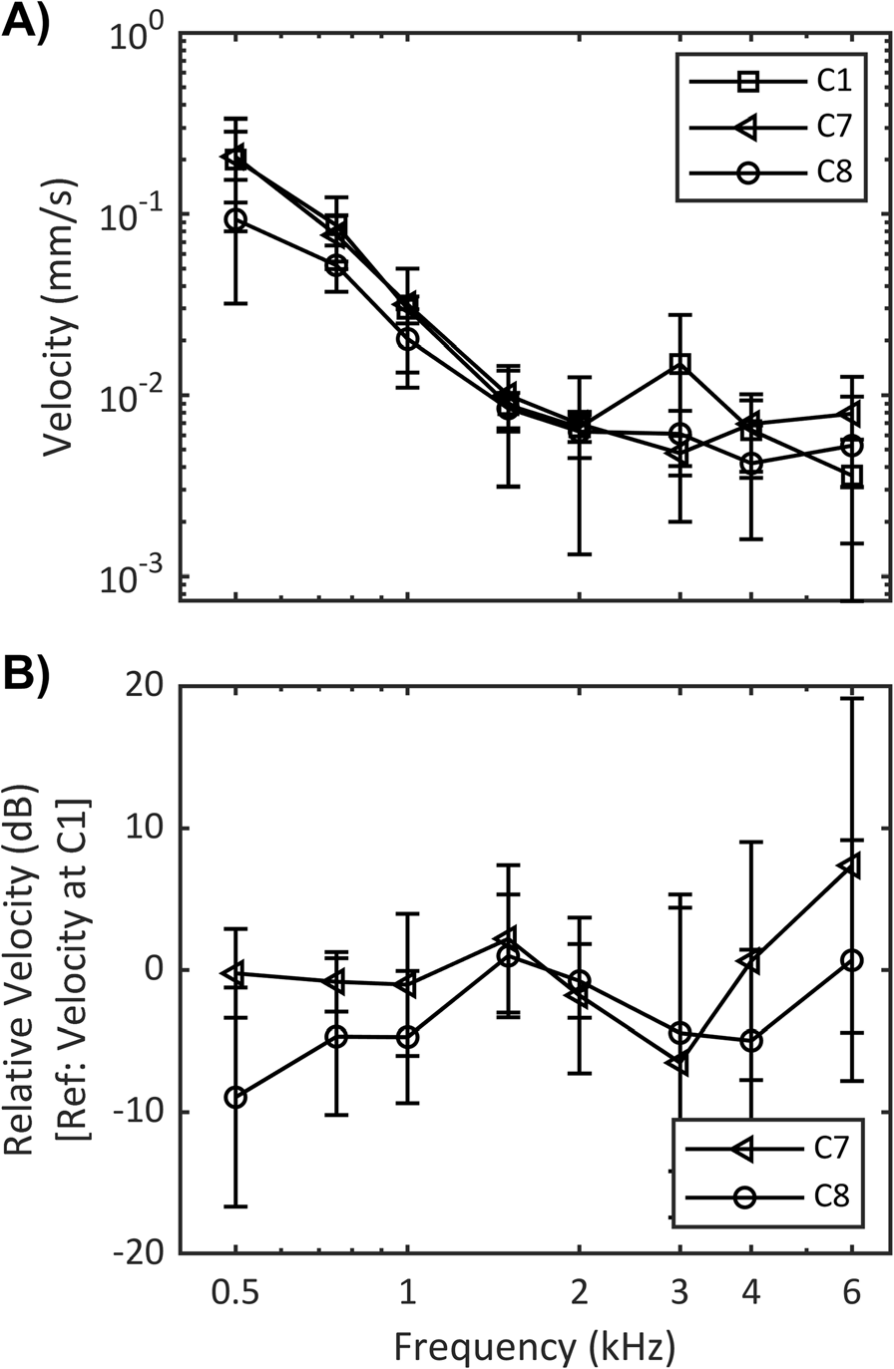
Effect of location with screw type and lift presence controlled. C1: measurement at MF, C7: measurement at TM, C8: measurement at RS. **a** Frequency response velocity. **b** Relative velocity. Average vibration velocity for 6 samples are shown and error bars denote one standard deviation

The effect of location on transmitted sound to the cochlea was assessed by comparing the vibration velocity when screw type and lift presence were controlled for by comparing C7 and C8 against C1 (Fig. 2).

**Fig. 3:**
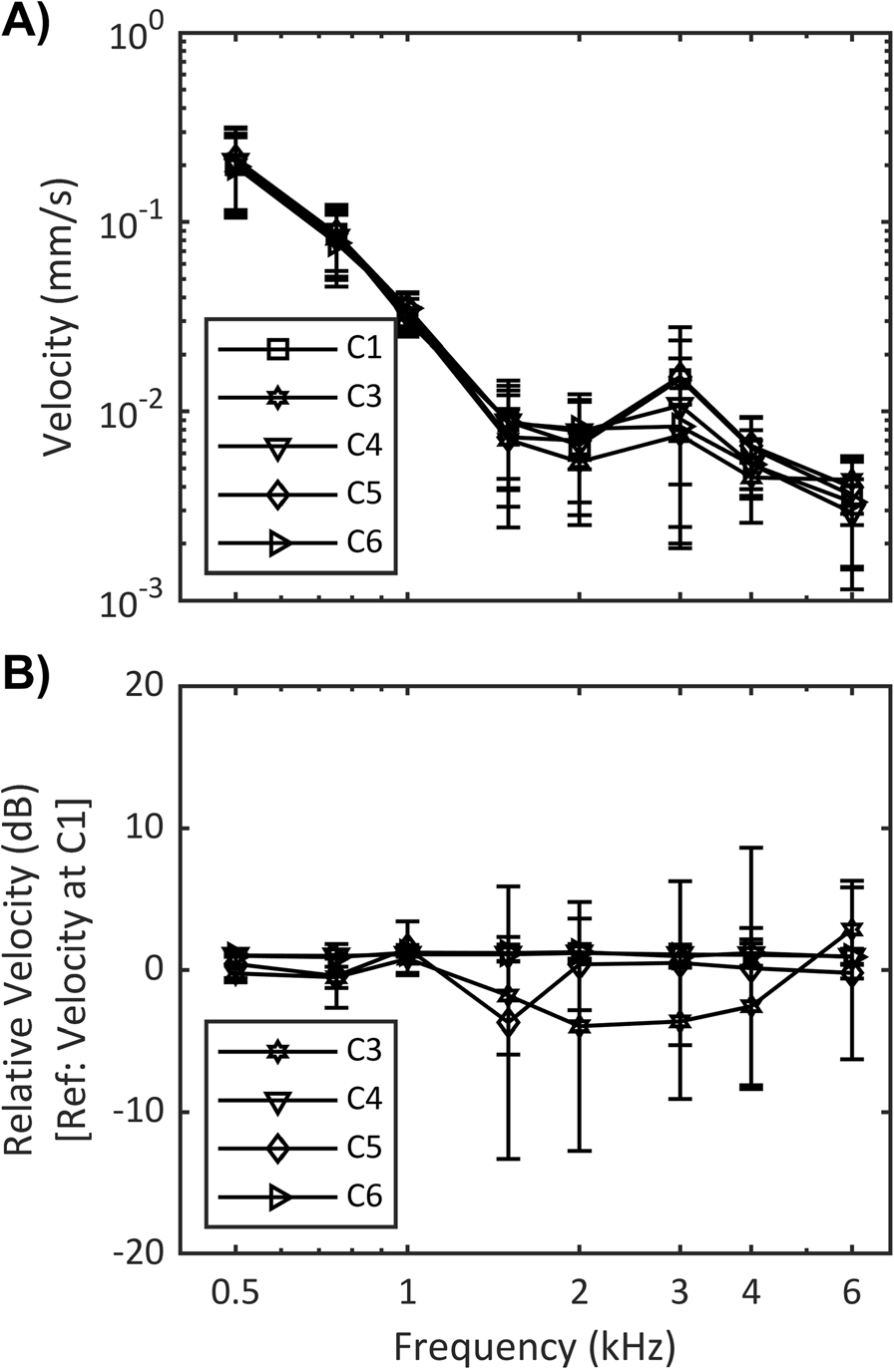
Effect of lift thickness with screw type and implant location controlled. C1: no lift, C3: 1 mm lift, C4: 2 mm lift, C5: 3 mm lift, C6: 4 mm lift. **a** Frequency response velocity. **b** Relative velocity. Average vibration velocity for 6 samples are shown and error bars denote one standard deviation

The effect of lift thickness on transmitted sound to cochlea was assessed by comparing the vibration velocity when the same type of screw and implant location were combined with various lift thicknesses (i.e., comparing C3-C6 against C1) as shown in Fig. 3.

**Fig. 4:**
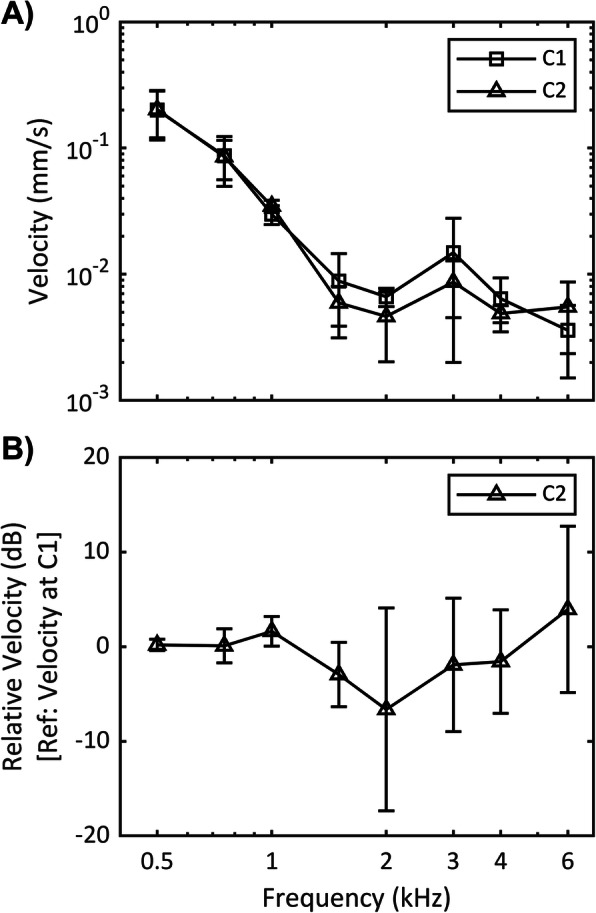
Effect of screw type with lift presence and implant location controlled. C1: self-tapping screw, C2: self-drilling screw. **a** Frequency response velocity. **b** Relative velocity. Average vibration velocity for 6 samples are shown and error bars denote one standard deviation

The effect of screw type on transmitted sound to cochlea was assessed by comparing the vibration velocity when location and lift presence was controlled for, but the screw type varied, i.e., comparing C2 against C1 (Fig. 4).

The one-way ANOVA did not find any statistically significant differences between any of the conditions at any of the test frequencies. The minimum *p*-value found was 0.2.

## Discussion

The BB-BCI is the first active, transcutaneous BCI and multiple studies have shown it to be an effective device to treat hearing loss in patients with mixed or conductive hearing loss [10–15]. The BB-BCI can be implanted under a variety of surgical techniques, with the implant housed in either the TM or RF, and recently the MF location. The MF location is associated with improvements such as reduced operating time and a smaller skin incision [1]. The MF approach was first performed at London Health Sciences Centre (LHSC), London, ON, Canada in 2013 [10] with favorable surgical results reported with a follow-up of up to 6 years [1]. Since then, other groups have also used the MF approach with or without self-drilling screws with clinical success [1]. At our centre (LHSC), self-drilling screws are used in place of the self-tapping screws included in the implant kit and lifts are used; however, there have not been any empirical studies of the effects of these modifications. The effects of modifications to the surgical approach, including implant location, lift usage and screw type, as three possible sources of variability on BB-BCI performance, were investigated in this study. Implant performance was assessed by measuring the vibration velocity at the cochlear promontory using an LDV technique.

The current study is the first to investigate the effects of common surgical variables on the performance of the BB-BCI; however, similar investigations have been published for other direct-drive systems. The effects of stimulation distance to the cochlea and squamosal suture on percutaneous BCI performance were investigated by Eeg-Olofsson et al. [9]. It was concluded that in the typical implant position a 10–20 dB lower response was seen compared to locations closer to the cochlea, and squamosal suture does not have a significant effect on the sound delivered. Rigato et al. studied the effect of implant attachment on the transmitted vibration to the cochlea using a balanced electromagnetic separation transducer [16]. The authors concluded that a smaller attachment might result in better performance at higher frequencies (above 5 kHz). In this study, the implant was placed in three surgical locations which had similar linear distances to the cochlea (30 to 40 mm). Therefore, if significant differences between locations had been found, they would likely have been secondary to bone thickness, suture lines, and/or screw fixation.

In studies focusing on the effect of surgical approach, it is important to only change one source of variability at a time. By using cadaveric temporal bones in the present study instead of intact cadaveric heads, we were able to fully control our experimental setup and testing. A total of eight distinct conditions (C1 – C8) were tested in each of six specimens. Since C1 had two out of three sources of variability in common with the remaining conditions, it was used as the reference condition. The mass of each temporal bone was constant at all conditions, since all three implant locations were drilled before measurements. Also, absence of brain matter precluded any additional source of variability when comparing implantations at the TM location compared to the MF and RS locations. In addition, pre-drilling of screw holes allowed for changing the location, lift and screw type with minimal movement, so the angle of the laser beam on the cochlear promontory could be kept constant in each sample. Lastly, the wired connection between the processor and the signal generator bypassed performance variabilities such as battery life and microphone-speaker angle.

One limitation of the present study was that only one combination of lift and screw type was used in each of the TM and RS locations. This was because the air cells in the mastoid limited the possibility of finding multiple suitable screw hole locations. Also, in the RS approach, the area behind the sigmoid sinus was too small to allow multiple screw holes.

The present findings did not reveal any significant differences associated with location, lifts or screw type. This allows surgeons the flexibility to choose the appropriate location and fixation for their particular clinical scenario without possible effects on sound transmission. We did not evaluate the potential effects of the amount of soft tissue removed on sound transmission, however since the BB-BCI is an active implant mounted directly to the bone via two screws, there should be minimal damping of sound caused by the overlying skin.

Although no significant differences were found between locations, placement of the BB-BCI in the MF location may be preferred as it causes less disruption of the air cells and nuchal musculature, requires a shorter operating time, and offers improved post-operative scar cosmesis [1, 11]. However, the most appropriate surgical approach should be determined by the primary surgeon depending on each patient’s anatomy and pathology. Further studies may benefit from analysis on fresh cadaveric samples or in-vivo measurements.

## Conclusion

The effects of implant location, lift thickness and screw type on BB-BCI were investigated on six cadaveric temporal bones using a LDV technique. This is the first known study to evaluate the effect of these variables on the sound transmission produced by the BB-BCI. No significant difference was found related to any of these modifications.

## Data Availability

Not applicable.

## Abbreviations

ANOVA: Analysis of variance
BB-BCI: BONEBRIDGE bone conduction implant
BCI: Bone conduction implant
LDV: Laser Doppler vibrometry
MF: Middle fossa
RS: Retrosigmoid
TM: Transmastoid
